# CT of Soft Tissue Infections: Current and Future Perspectives from Diagnosis to Treatment

**DOI:** 10.3390/jcm15103915

**Published:** 2026-05-19

**Authors:** Federico Pistoia, Marta Macciò, Riccardo Picasso, Federico Zaottini, Maria Elena Susi, Giovanni Marcenaro, Carlo Martinoli

**Affiliations:** 1 IRCCS Azienda Ospedaliera Metropolitana, 16132 Genoa, Italy; federico.pistoia@aomliguria.it (F.P.);; 2 Department of Health Sciences (DISSAL), University of Genoa, 16132 Genoa, Italy; 3 Department of Experimental Medicine (DIMES), University of Genoa, 16132 Genoa, Italy

**Keywords:** soft-tissue infections, imaging CT, emergency department

## Abstract

**Background/Objectives**: Skin and soft-tissue infections represent a critical diagnostic challenge in the emergency department, requiring rapid differentiation between superficial processes and life-threatening necrotizing conditions. **Methods**: This clinical update analyzes the central role of CT in the assessment of these pathologies, proposing a management-oriented algorithm to support risk stratification and therapeutic planning. **Results**: By reviewing the fundamental semiological features of contrast-enhanced CT, this work highlights how the identification of critical markers directly guides the choice between medical management and urgent surgical intervention. In addition to consolidating protocols based on conventional CT, the article examines current perspectives and clinical limitations of emerging technologies, including Spectral CT (DECT and PCD-CT) and artificial intelligence, evaluating their potential to improve image quality and tissue characterization. **Conclusions**: In conclusion, while technological evolution offers new diagnostic frontiers, a rigorous and systematic interpretation of CT findings remains the fundamental pillar for optimizing patient outcomes and reducing unnecessary interventions.

## 1. Introduction

Skin and soft-tissue infections (SSTIs) are a common reason for presentation to the emergency department (ED) and include a spectrum of non-purulent, purulent, and necrotizing conditions. Imaging plays a pivotal role in the evaluation of these infections, especially when clinical assessment is limited by pain, swelling, or nonspecific findings. In this setting, imaging helps differentiate superficial processes from deep or necrotizing disease and supports timely and appropriate management decisions. The main contribution of radiography lies in the detection of soft tissue gas, foreign bodies, or associated osseous abnormalities, findings that may raise suspicion for necrotizing infection but lack sufficient sensitivity to exclude it [1,2]. Ultrasound (US) is commonly used as the first-line imaging modality for clinically superficial soft tissue infections. US allows detection of superficial abscesses, differentiation between cellulitis and fluid collections, and real-time guidance for percutaneous drainage, without ionizing radiation. Nevertheless, US is operator-dependent and limited in evaluating deep compartments, and fascial planes, particularly in obese patients or in the presence of extensive soft tissue edema. Although MRI offers excellent soft tissue contrast and high sensitivity for fascial and muscular involvement, its limited availability, longer acquisition times, and frequent incompatibility with unstable or critically ill patients restrict its use in the emergency setting, and it is therefore not considered in this review [2]. In this diagnostic pathway, contrast-enhanced CT (CECT) represents the cornerstone imaging modality for suspected deep or complicated soft tissue infections. CT provides rapid, comprehensive evaluation of large anatomic regions and enables differentiation among cellulitis, phlegmon, abscess, fasciitis, necrotizing fasciitis, and pyomyositis by depicting fascial involvement, intramuscular extension, gas formation, and multicompartmental spread. These imaging features directly influence clinical management, particularly the need for urgent surgical exploration, image-guided drainage, or escalation of antimicrobial therapy. The purpose of this narrative review is to describe the role of CT in the assessment of soft tissue infections in the ED, with emphasis on imaging findings that impact management and common diagnostic pitfalls. While previous reviews have addressed the imaging spectrum of soft tissue infections in the broader musculoskeletal context, the present work differs in three respects. First, it is specifically oriented toward the emergency setting, where time constraints, patient instability, and limited access to MRI often make CT the de facto primary diagnostic tool. Second, it proposes a structured, visually explicit management algorithm that integrates clinical risk stratification with imaging triage, offering a practical tool for clinical decision-making. Third, it incorporates the most recent evidence to provide an updated perspective on traditional diagnostic scores, the current indications for CT, and a realistic assessment of emerging technologies, such as spectral CT and artificial intelligence, in this clinical context. Diagnostic performance figures cited throughout this review are derived from individual studies with heterogeneous designs, patient populations, and reference standards; they should not be interpreted as pooled estimates and must be considered with appropriate caution.

**Literature Search.** The literature for this narrative review was identified through searches of PubMed, conducted without date restrictions and last updated in January 2026 (Table 1). Search queries included the following terms and their combinations: “soft tissue infection CT,” “necrotizing fasciitis computed tomography,” “skin and soft tissue infection imaging emergency,” “cellulitis CT diagnosis,” “pyomyositis imaging,” “Fournier gangrene CT,” “dual-energy CT infection,” “photon-counting CT musculoskeletal,” “artificial intelligence necrotizing soft tissue infection,” and “LRINEC score validation.” Reference lists of identified articles were manually screened to retrieve additional relevant publications. No formal search protocol was pre-registered, no predefined inclusion or exclusion criteria were applied, and no systematic quality appraisal of individual studies was performed. Article selection was based on clinical and methodological relevance to the review topics as assessed by the authors. These characteristics are inherent to the narrative review format and constitute acknowledged limitations, as discussed in Section 11. The SANRA Checklist is provided in the Supplementary Materials as Table S1.

## 2. Cellulitis and Phlegmon

**Figure 1:**
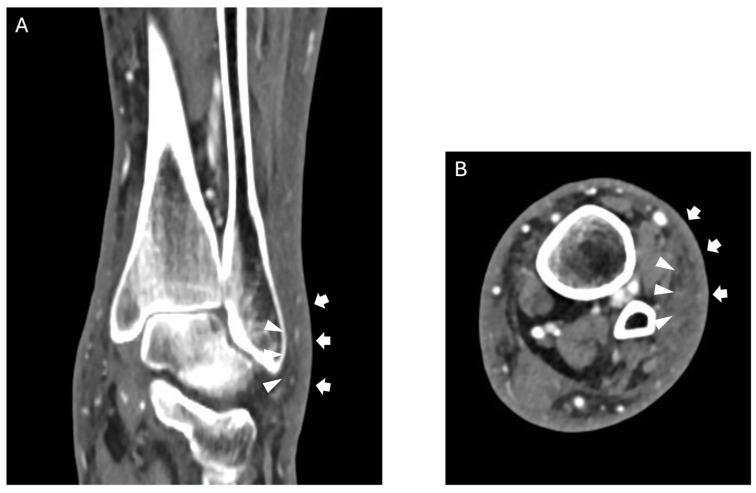
Coronal (**A**) and axial (**B**) CT images show dermal thickening (arrows) and soft tissue edema (arrowheads) consistent with cellulitis. Note on figures: Figure 1, Figure 2, Figure 3 and Figure 4 are representative educational cases selected from an anonymized institutional archive and they are intended as illustrative examples to support the descriptive text.

Cellulitis is an infection limited primarily to the dermis and subcutaneous fat, typically presenting with erythema, warmth, swelling, and pain. In many cases, the diagnosis is clinical, and imaging is not required [1,2]. When performed, CT demonstrates skin thickening, increased attenuation of subcutaneous fat with a reticular or strandy appearance, and sometimes thickening of superficial fascia without a discrete fluid, early venous return, and lymph node enlargement [2,11,12,13] (Figure 1). Phlegmon represents a more advanced inflammatory process with confluent soft tissue edema, but still lacks a well-formed, drainable collection. On CECT, these regions show diffuse, ill-defined enhancement rather than a sharply marginated rim [2,11]. These findings are inherently nonspecific and can overlap with noninfectious edema or inflammatory conditions, so correlation with clinical features and laboratory markers is essential [2,11,14]. Importantly, recent work in extremity cellulitis shows that routine CT has a low yield for detecting deep infection (approximately 5–8%), and imaging should be reserved for high-risk presentations or when deeper involvement is suspected [8,9]. Therefore, the routine use of CT in uncomplicated cellulitis is generally not recommended due to its limited clinical impact [8,9].

## 3. Fasciitis and Necrotizing Fasciitis

**Figure 2:**
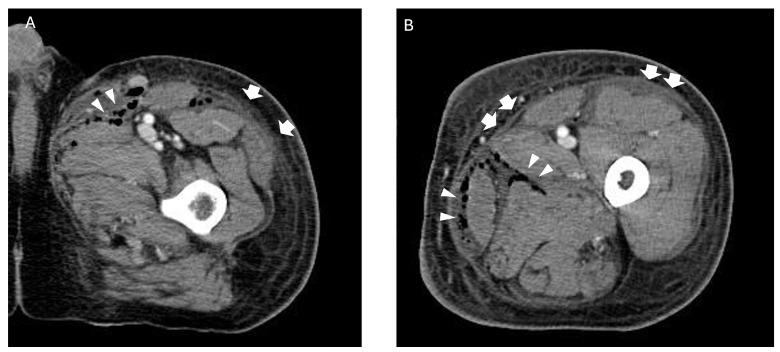
Axial CT images (**A**,**B**) of lower extremity show soft tissue edema, thickening fascial (arrowheads) and internal gas (arrows) tracking along superficial and deep fascial planes, consistent with necrotizing fasciitis.

**Figure 3:**
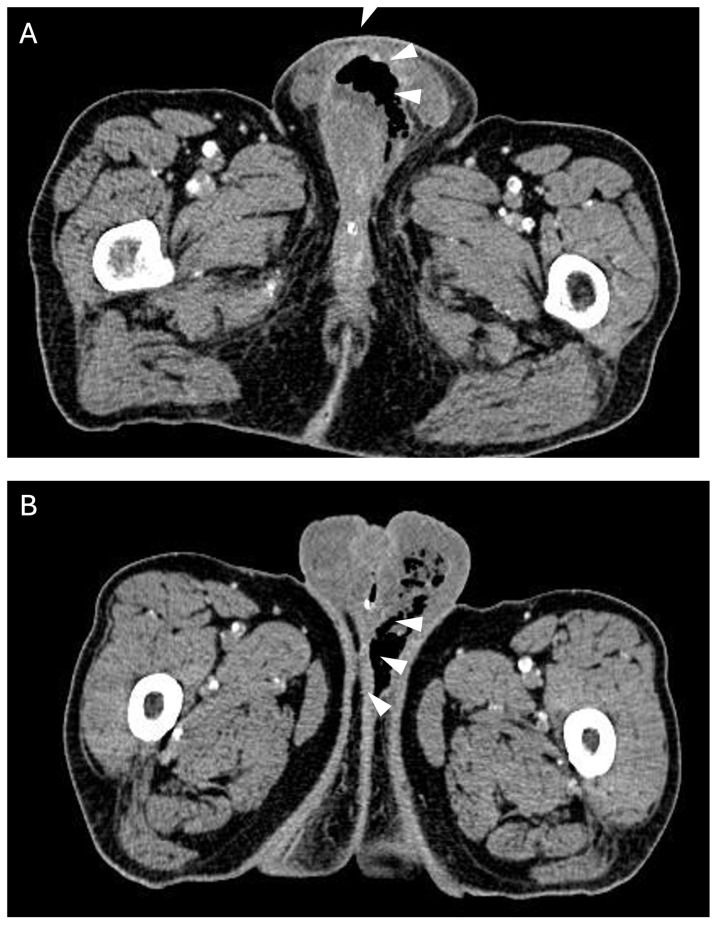
Axial CT images (**A**,**B**) show extensive soft tissue stranding with edema in penis and scrotum and a large volume of gas air (arrowheads) tracking into deep soft tissues of perineum representing Fournier gangrene.

Fasciitis refers to inflammation of the fascia, the connective tissue enveloping muscles, vessels, and nerves. This condition may result from a wide range of infectious agents, including bacteria, fungi, viruses, and parasites, and it can in severe cases progress rapidly to tissue necrosis, as observed in necrotizing fasciitis (NF). Necrotizing fasciitis is a fulminant, life-threatening bacterial infection characterized by extensive fascial necrosis, systemic toxicity, and high mortality if not promptly treated [15]. Early clinical manifestations of fasciitis and NF often overlap and include pain, swelling, and erythema. However, NF typically presents with pain disproportionate to physical findings and may be associated with bullae, skin discoloration, or soft-tissue gas, features that can aid clinical suspicion [16]. Unlike non-necrotizing fasciitis, which may respond to medical therapy, NF requires urgent surgical debridement in addition to broad-spectrum antibiotics and intensive supportive care [15,16]. The Laboratory Risk Indicator for Necrotizing Fasciitis (LRINEC) score is a commonly used clinical tool designed to differentiate NF from other soft-tissue infections using six routine laboratory parameters: white blood cell count, hemoglobin, sodium, glucose, creatinine, and C-reactive protein [5]. Scores ≥ 6 suggest an increased likelihood of NF. While early studies reported high predictive value, subsequent validations have demonstrated variable sensitivity and specificity, particularly in ED settings, where false-negative results may occur [17,18,19]. Despite these limitations, the LRINEC score retains prognostic value, as higher scores have been associated with longer hospital stays, increased ICU admission, and higher mortality [20]. Several modified or combined scoring systems incorporating additional clinical parameters have been proposed to enhance diagnostic accuracy [21,22]. However, imaging, particularly CT evidence of deep fascial fluid, has been shown to outperform LRINEC alone in diagnosing NF, and combined use of clinical, laboratory, and imaging data provides the greatest diagnostic confidence [4] (Figure 2). Machine learning models using clinical and laboratory data also show potential for improving early NSTI diagnosis and may play a complementary role alongside imaging, although they are not yet part of standard practice [23,24]. Characteristic CT findings include asymmetric fascial thickening, fat stranding, blurring of fascial planes, and gas tracking along fascial planes, reported in more than half of cases [6,25]. The presence of fluid collections along the deep fascia is considered highly suggestive of NF and has been shown to be more diagnostically useful than laboratory-based scoring systems alone [26]. Additional features such as soft-tissue air and inflammatory changes extending into adjacent muscles or compartments further support the diagnosis and help distinguish NF from less severe soft-tissue infections [26]. On CECT, non-enhancement of the fascia is regarded as a strong indicator of tissue necrosis, reflecting compromised perfusion. Although this finding is not pathognomonic in isolation, its presence in combination with asymmetric fascial involvement significantly increases diagnostic confidence. CT is also valuable in assessing disease extent, including multicompartmental involvement and spread to adjacent regions, thereby aiding surgical planning [26]. A notable subtype of necrotizing fasciitis is Fournier gangrene, a rapidly progressive infection involving the perineal, perianal, and genital regions, predominantly affecting men aged 50–70 years, often with diabetes mellitus. In this context, CT is particularly valuable for delineating the extent of involvement of the genitalia, perineum, ischiorectal fossae, abdominal wall, or retroperitoneum, findings that are critical for surgical planning and prognostication [27,28,29] (Figure 3). Interobserver agreement for CT findings in NF is generally good, supporting its reliability in clinical practice; however, reproducibility varies by finding type. Objective binary signs, such as the presence of soft tissue gas and fluid collections along fascial planes, tend to show higher interobserver agreement, whereas more subjective assessments, including the degree of fascial non-enhancement, subtle asymmetric fascial thickening, and blurring of fascial planes in early-stage disease, are more susceptible to variability related to acquisition quality, contrast timing, and reader experience [28]. Emerging data suggest that volumetric CT assessment of air and soft-tissue involvement may correlate with hospital length of stay and healthcare costs, although no single CT parameter has been shown to reliably predict mortality [10]. Overall, CT demonstrates high diagnostic performance, with reported sensitivity of approximately 88.5% and specificity of 93.3% for NF [1,4].

## 4. Cellulitis vs. Fasciitis on CT

On CT imaging, the differentiation between cellulitis and fasciitis primarily hinges on the involvement and appearance of the fascial planes. Cellulitis typically presents as diffuse thickening and infiltration of the skin and subcutaneous tissues with fat stranding but without deep fascial involvement or fluid collections [26]. In contrast, fasciitis and NF show more specific features, including diffuse thickening and enhancement of the superficial and deep fasciae, fluid collections along the fascial planes, and sometimes gas within the soft tissues [26,30]. CT can also reveal muscle involvement (myositis) and abscess formation in fasciitis, which are less common in uncomplicated cellulitis [26].

## 5. Soft Tissue Abscess

Soft tissue abscesses are localized collections of pus within the skin or soft tissues that require accurate differentiation from cellulitis because their treatments differ significantly. On CT imaging, soft tissue abscesses typically appear as fluid collections with low attenuation surrounded by a rim of contrast enhancement, which corresponds to the abscess capsule. While CT is more specific, it is less sensitive than US for detecting superficial soft tissue abscesses, since US generally provides superior visualization of the abscess cavity and internal details [31]. Point-of-care ultrasonography has demonstrated high diagnostic accuracy, with sensitivity around 93–98% and specificity between 85 and 92%, making it a valuable tool in emergency settings to distinguish abscesses from cellulitis and guide management decisions [7,32,33,34]. Overall, US is preferred for initial evaluation of superficial abscesses due to its sensitivity and detailed imaging, while CT serves as complementary modalities, especially for complex or deep abscesses [2,11].

## 6. Pyomyositis

**Figure 4:**
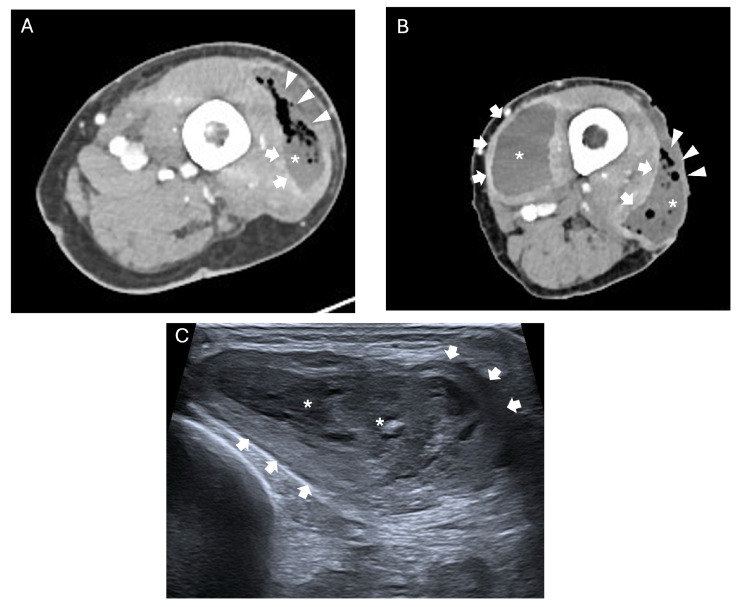
Axial contrast-enhanced CT (**A**,**B**) and long-axis ultrasound (**C**) images of the lower extremity demonstrate the same intramuscular lesion consistent with pyomyositis. On CT it appears as a fluid collection (asterisks) with marked peripheral rim enhancement (arrows) and intralesional gas locules (arrowheads). On ultrasound, a corresponding heterogeneously hypoechoic intramuscular collection (asterisks) with internal debris and a surrounding echogenic capsule (arrows) is observed.

Pyomyositis is a primary bacterial infection of skeletal muscle that most commonly affects immunocompromised patients, individuals with diabetes mellitus, and populations in tropical climates, although it is increasingly recognized worldwide [2,11]. Clinically, patients typically present with fever and localized muscle pain and swelling; however, early manifestations may be subtle and laboratory findings nonspecific, making imaging important for early diagnosis and for differentiating pyomyositis from traumatic muscle injury, diabetic muscle infarction, or neoplasm [2,11,14]. From both a pathophysiological and imaging standpoint, pyomyositis represents a spectrum of disease rather than a single static entity. On CT, early-stage pyomyositis manifests as enlargement of the affected muscle with decreased attenuation and heterogeneous enhancement, reflecting edematous and inflamed muscle without a well-formed collection [2,11]. As the infection progresses to the suppurative stage, intramuscular low-attenuation collections develop, surrounded by peripheral rim enhancement consistent with abscess formation; adjacent soft-tissue edema and overlying cellulitis are common. In this context, the intramuscular abscess should be regarded as the imaging expression of advanced pyomyositis rather than a distinct radiological entity [2,11]. Intramuscular gas is less frequent than in necrotizing fasciitis but may occasionally be present (Figure 4). In contrast, diabetic muscle infarction typically demonstrates poor or absent enhancement and lacks a drainable collection, whereas pyomyositis usually shows focal areas of enhancement and, in later stages, a well-defined abscess cavity [14,35]. Recognition of these evolving imaging patterns is important, as it facilitates prompt initiation of antibiotic therapy and timely percutaneous or surgical drainage, thereby reducing the risk of systemic sepsis and long-term complications [2,11,35]. The CT features and management strategies of soft tissue infections are summarized in Table 2.

## 7. CT vs. US

**Figure 5:**
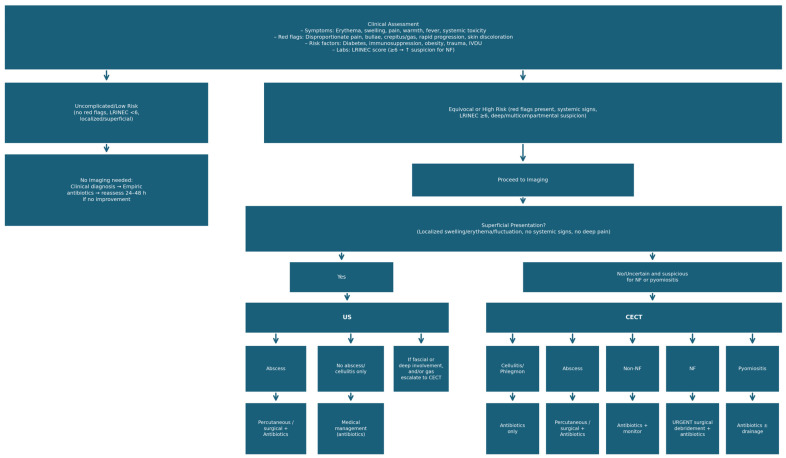
Proposed diagnostic and management algorithm for suspected soft tissue infections.

A common diagnostic dilemma in the ED regards the choice of the correct first imaging modality to study soft tissue infections. While radiography has intrinsic limitations in the assessment of soft-tissue, US represents a viable alternative to CT in some settings. Usually, in cases of clinically superficial soft tissue infection characterized by localized swelling, erythema, fluctuation, and absence of systemic signs, US is the recommended first-line imaging to detect abscesses and guide drainage if needed [36]. If no abscess is detected, conservative management is appropriate. However, when clinical features suggest higher risk, such as severe pain, sepsis, rapid progression, or immunosuppression, CECT is indicated to assess the depth, extent, fascial involvement, presence of gas, and complications, which are critical for differentiating necrotizing soft tissue infections from simple cellulitis and guiding surgical versus medical management [30,36,37]. The diagnostic performance of US is substantially influenced by operator experience, patient body habitus, and depth of infection; in centres without dedicated emergency sonography expertise, reproducibility cannot be assumed and CT may represent a more reliable first-line choice. The diagnostic workflow for suspected soft tissue infections is illustrated in Figure 5. It should be noted that this algorithm is conceptual and expert opinion-based; it has not been prospectively validated, and its impact on clinical decisions or patient outcomes has not been formally studied.

## 8. DECT, PCD-CT, and AI: Current Perspectives and Clinical Limitations

Dual-energy CT (DECT) improves soft tissue infection imaging by exploiting material-dependent attenuation differences at two X-ray energy spectra, enabling tissue characterization beyond conventional single-energy CT. By generating virtual monochromatic images and material-specific maps, particularly iodine maps, DECT enhances lesion conspicuity and depicts inflammation-related perfusion changes [38,39,40]. Low-keV virtual monochromatic reconstructions (approximately 40–50 keV) significantly increase soft-tissue contrast, facilitating discrimination between different soft tissues. In addition, DECT can reduce beam-hardening artifacts and improve overall image quality, which may aid diagnostic confidence [41,42]. Although DECT has been widely investigated in musculoskeletal and oncologic imaging, its application to soft tissue infections remains comparatively underexplored, and dedicated clinical studies are still limited. Consequently, further research is required to define standardized protocols and validate its incremental diagnostic value in this setting [42]. Photon-counting detector CT (PCD-CT) represents a newer technological advance, offering improved spatial resolution, enhanced contrast-to-noise ratio, and intrinsic spectral imaging compared with conventional CT systems [43,44,45]. These features enable superior visualization of soft tissues and more accurate material differentiation, which may theoretically improve assessment of infection-related changes. Early clinical experience has demonstrated improved depiction of soft tissue edema and inflammatory changes in noninfectious conditions, such as acute musculoskeletal trauma [46]. Quantitative iodine mapping with PCD-CT further allows differentiation of inflamed tissue from surrounding structures, supporting disease activity assessment in inflammatory processes and suggesting potential applicability to infection imaging [47]. However, direct evidence evaluating the diagnostic accuracy of PCD-CT specifically for soft tissue infections, or comparing it with conventional CT and MRI, is currently lacking. Overall, any meaningful improvement in the diagnosis of soft tissue infections with DECT or PCD-CT compared with conventional CT has not yet been demonstrated, and their practical role in emergency imaging remains theoretical. In parallel with hardware-based advances, automated CT analysis using artificial intelligence has emerged as a potential tool for detecting necrotizing soft tissue infection through pattern recognition of key imaging features, including fascial edema, fluid tracking, soft tissue gas, and abnormal enhancement. However, similar to DECT and PCD-CT, these approaches currently lack robust prospective validation and should therefore be regarded as investigational. Current AI studies in this domain are further limited by small dataset sizes, lack of external validation, risk of overfitting, and potential spectrum bias, all of which restrict generalizability to routine emergency imaging practice.

## 9. Diagnostic Pitfalls and Mimics

Accurate CT interpretation of soft tissue infections requires careful differentiation between infectious and non-infectious conditions, as the presence of soft tissue gas—although highly specific—is not pathognomonic for necrotizing fasciitis (NF). Iatrogenic air after surgery or trauma may track along fascial planes and closely mimic NF. Conversely, systemic edematous states such as anasarca or congestive heart failure can produce diffuse subcutaneous fat stranding and fascial thickening. In these settings, bilateral symmetric involvement and the absence of systemic toxicity should raise suspicion for non-infectious etiologies [3,48]. A major diagnostic limitation is the low sensitivity of gas on CT, which is absent in approximately 30–50% of confirmed NF cases. This is particularly common in monomicrobial type II infections, such as those caused by Streptococcus pyogenes [3,47]. Over-reliance on gas as a primary diagnostic sign therefore increases the risk of false-negative interpretation. Instead, radiologists should prioritize more sensitive indicators, notably asymmetric deep fascial fluid and fascial non-enhancement on contrast-enhanced CT. Fascial non-enhancement is especially critical, as it represents a direct imaging correlate of fascial necrosis—a surgical emergency requiring immediate intervention [49]. The reliability of these imaging findings depends heavily on optimized acquisition protocols and awareness of technical pitfalls. Incorrect contrast timing is a key issue: scans obtained in the early arterial phase may falsely suggest absent fascial enhancement. Assessment of tissue viability is best performed during the portal venous phase, approximately 60–80 s after contrast administration. In patients with metallic hardware, beam-hardening artifacts may obscure adjacent soft tissues; in such cases, dual-energy CT and metal artifact reduction algorithms can substantially improve diagnostic evaluation [50]. To minimize technical heterogeneity and support reproducible image interpretation, a standardized acquisition protocol is recommended. Scan coverage should encompass the entire clinically involved region, with extension to the chest, abdomen, or retroperitoneum when fascial spread is suspected, as necrotizing infection may track along fascial planes into deep compartments [51,52]. Although no soft tissue infection study defines a consistent standardized contrast timing protocol, a venous-phase acquisition with a field of view tailored to the affected region, as routinely used in general CT practice, is the pragmatic choice that best supports assessment of fascial enhancement, and abscess walls. Images should be reconstructed at ≤3 mm slice thickness using a soft-tissue kernel, with additional thin-section reconstructions at approximately 1–1.25 mm for multiplanar review.

## 10. Resource and Cost Implications

A formal cost-effectiveness analysis was beyond the scope of this review, and no dedicated economic studies on CT use in soft tissue infections were identified in the literature. Nevertheless, several practical considerations merit acknowledgment. CT involves ionizing radiation, iodinated contrast administration, with nephrotoxicity risk in patients with pre-existing renal impairment or other predisposing factors [53] and allergic risks, and non-negligible costs, all of which should inform its use in clinical decision-making. The selective approach advocated throughout this review, reserving CT for high-risk presentations and avoiding routine use in uncomplicated cellulitis [9], is consistent not only with diagnostic appropriateness but also with resource stewardship. Furthermore, access to CT, availability of experienced radiologists, and emergency workflow organization vary substantially across institutions and healthcare systems, which may limit the generalizability of the proposed diagnostic approach to all clinical settings. Future research should include prospective studies evaluating the clinical and economic impact of CT-based triage algorithms in soft tissue infections, including effects on time to surgery, rates of negative surgical exploration, and hospital resource utilization.

## 11. Limitations

As inherent to the narrative review format, several limitations apply to this work. The absence of a systematic search strategy, predefined inclusion criteria, and formal quality appraisal of included studies means that selection bias cannot be excluded and that evidence from studies of varying methodological strength is presented without explicit grading. The reported diagnostic performance figures for CT derive from heterogeneous studies and should be interpreted with caution, as they have not been pooled using meta-analytic methods. Clinical and technical heterogeneity, including variation in patient comorbidities, CT acquisition protocols, and reference standards, further limits the generalizability of individual estimates. The review focuses on imaging findings rather than patient-centred outcomes, and the proposed diagnostic algorithm (Figure 5) is conceptual and not yet prospectively validated. The sections on dual-energy CT, photon-counting detector CT, and artificial intelligence reflect an early evidence base and should be regarded as investigational. Finally, as discussed above, a formal cost-effectiveness analysis was beyond the scope of this review, and the generalizability of the proposed approach may vary substantially across healthcare settings with different levels of CT access and radiological expertise.

## 12. Conclusions

In the ED, CECT is the key imaging modality for evaluating suspected deep or complicated SSTIs, particularly when clinical findings are equivocal or disease severity must be rapidly stratified. US remains appropriate for superficial infections, while emerging spectral CT technologies may further refine tissue characterization but are not yet integral to routine emergency imaging practice. Accurate interpretation of imaging findings, combined with clinical and laboratory data, remains essential to avoid both delayed diagnosis of necrotizing infection and unnecessary intervention.

## Figures and Tables

**Table 1:** Summary of key studies reviewed.

Study	Design	Population	Key Variable Assessed	Main Finding
Fernando et al., 2019 [1]	Systematic review and meta-analysis	Suspected NSTI (25 studies)	CT diagnostic accuracy for NF	CT sensitivity 88.5%, specificity 93.3%
Kwee & Kwee, 2022 [3]	Systematic review	Suspected NF (11 studies)	MRI vs. CT diagnostic accuracy	Both modalities show high accuracy; CT preferred for wider emergency availability
Bruls & Kwee, 2021 [4]	Retrospective cohort	35 confirmed NF cases	CT criteria vs. LRINEC score	CT outperforms LRINEC; deep fascial fluid most diagnostically useful CT finding
Wong et al., 2004 [5]	Prospective cohort	89 NF, 225 non-NF patients	LRINEC score development and validation	Sensitivity 80%, specificity 96% at cutoff ≥6; variable performance in subsequent validations
Wysoki et al., 1997 [6]	Retrospective	16 NF cases	CT characteristics of NF (gas, fluid, fascial involvement)	Gas present in 55% of NF; fascial fluid in 100%; CT superior to plain radiography
Gottlieb et al., 2020 [7]	Meta-analysis	Skin and soft tissue abscesses (10 studies)	Point-of-care US diagnostic accuracy	Sensitivity 93–98%, specificity 85–92%; POCUS superior to CT for superficial abscesses
Burke et al., 2025 [8,9]	Retrospective cohort	ED patients with extremity cellulitis	CT yield for deep infection and CT utilization patterns	Deep infection identified in ~5–8% of CT examinations; CT not recommended routinely for uncomplicated cellulitis
Ganapathy et al., 2024 [10]	Retrospective cohort	Fournier’s gangrene patients	Volumetric CT measurements as outcome predictors	Volumes of gas and soft tissue involvement correlate with hospital length of stay and healthcare costs

NSTI, necrotizing soft tissue infection; NF, necrotizing fasciitis; ED, emergency department; LRINEC, Laboratory Risk Indicator for Necrotizing Fasciitis; US, ultrasound; POCUS, point-of-care ultrasonography.

**Table 2:** CT imaging features and management implications of soft tissue infections. The management implications listed reflect narrative synthesis and expert interpretation; they are intended to contextualize imaging findings within clinical management rather than serve as formal evidence-graded recommendations.

Entity	CT Features	Management Implications
Cellulitis/Phlegmon	Skin/subcutaneous thickening, fat stranding/reticular pattern, ill-defined enhancement; no discrete collection or deep fascia involvement.	Medical management; antibiotics. CT not routinely indicated in uncomplicated cases. Reserve imaging for high-risk presentations or diagnostic uncertainty.
Abscess	Low-attenuation fluid collection with rim enhancement; may have gas/debris.	Percutaneous or surgical drainage + antibiotics. US preferred for superficial abscesses; CT for deep or complex collections.
Fasciitis (non-necrotizing)	Fascial thickening/enhancement, fluid along fascia; no necrosis/non-enhancement.	Antibiotics + close clinical monitoring. Serial imaging if no improvement. Surgical exploration if progression to necrosis suspected.
Necrotizing Fasciitis (NF)	Asymmetric fascial thickening, blurring/gas along fascia, non-enhancing fascia (necrosis), multicompartment spread, muscle involvement; fluid collections highly suggestive. Subtype: Fournier’s (perineal extension).	URGENT surgical debridement + broad-spectrum antibiotics + ICU. Immediate surgical consultation; do not delay for additional imaging.
Pyomyositis	Muscle enlargement, heterogeneous enhancement, low-attenuation intramuscular collections (abscess); adjacent edema/cellulitis.	Antibiotics + drainage (percutaneous or surgical) of intramuscular abscess when present. Early-stage disease may respond to antibiotics alone.

## Data Availability

The images supporting the conclusions of this article will be made available by the authors on request.

## Supplementary Materials

The following supporting information can be downloaded at: https://www.mdpi.com/article/10.3390/jcm15103915/s1, Table S1: SANRA Checklist.

## Abbreviations

AI	Artificial Intelligence
CECT	Contrast-Enhanced Computed Tomography
CT	Computed Tomography
DECT	Dual-Energy Computed Tomography
ED	Emergency Department
ICU	Intensive Care Unit
keV	Kiloelectronvolt
LRINEC	Laboratory Risk Indicator for Necrotizing Fasciitis
MRI	Magnetic Resonance Imaging
NF	Necrotizing Fasciitis
NSTI	Necrotizing Soft Tissue Infection
PCD-CT	Photon-Counting Detector Computed Tomography
SANRA	Scale for the Assessment of Narrative Review Articles
SSTIs	Skin and Soft-Tissue Infections
US	Ultrasound
