# Supplementary material for: CT of Soft Tissue Infections: Current and Future Perspectives from Diagnosis to Treatment
Source: J Clin Med. 2026 May 19;15(10):3915. doi: 10.3390/jcm15103915 (PMC13207358; doi:10.3390/jcm15103915)
Supplement: Supplementary file 1 [file jcm-15-03915-s001.zip › jcm-4256356-supplementary.pdf]

Table S1 – SANRA Checklist

Item	Criterion	Description	Score (0–2)	Location in manuscript	Justification
1	**Justification of the review topic's importance**	*Did the author(s) justify the importance of the review topic?*	**2**	Section 1 – Introduction	The Introduction clearly frames SSTIs as a common and diagnostically challenging ED presentation, establishes CT as the practical first-line modality, and identifies three specific gaps addressed by this review: emergency focus, a structured management algorithm, and an updated assessment of emerging technologies.
2	**Literature search**	*Did the author(s) describe their search method?*	**1**	Section 11 – Limitations	No systematic search strategy was employed, consistent with the narrative review format. The absence of a pre-specified search strategy and the resulting possibility of selection bias are explicitly acknowledged as inherent limitations in the dedicated Limitations section (Section 11).
3	**Appropriate referencing**	*Did the author(s) reference their data well?*	**2**	Throughout; Introduction revised in this version	Fifty-four references are cited throughout the manuscript, covering all major claims across sections. Following reviewer feedback, citations were added to the Introduction to support statements on plain radiography's role in the ED and MRI's logistical limitations in the emergency setting.
4	**Substantiation of assertions**	*Did the author(s) substantiate their assertions?*	**2**	Introduction (disclaimer); Section 11 – Limitations	All major assertions are supported by cited literature or explicitly caveated. A general disclaimer was added at the end of the Introduction: diagnostic performance figures are derived from heterogeneous individual studies and should not be interpreted as pooled estimates. This is reinforced in the Limitations section.

5	**Discernment**	*Did the author(s) distinguish between evidence, inference, and expert opinion?*	**2**	Section 7; Figure 5 caption; Table 1 caption; Section 11	The revised manuscript consistently distinguishes evidence-based statements from expert interpretation. Figure 5 is explicitly labeled as conceptual and expert opinion-based, not prospectively validated. Table 1 caption clarifies that management implications reflect narrative synthesis rather than formal evidence-graded recommendations. Language revisions removed overstatements throughout (e.g., 'essential' replaced by 'particularly valuable').
6	**Clarity of language**	*Was the language used clear for the readers?*	**2**	Throughout	The manuscript is written in clear and accessible scientific English, with a logical structure progressing from basic entities (cellulitis, abscess) to complex conditions (necrotizing fasciitis, pyomyositis) and emerging technologies. Terminology is consistent and appropriate for the target readership.
**Total**			**11 / 12**		One point deducted for Item 2: absence of a formal search strategy, inherent to the narrative review format and explicitly acknowledged in the Limitations section.
